# Analysis of the Role of Stellate Cell VCAM-1 in NASH Models in Mice

**DOI:** 10.3390/ijms24054813

**Published:** 2023-03-02

**Authors:** Kyoung-Jin Chung, Aigli-Ioanna Legaki, Grigorios Papadopoulos, Bettina Gercken, Janine Gebler, Robert F. Schwabe, Triantafyllos Chavakis, Antonios Chatzigeorgiou

**Affiliations:** 1 Institute for Clinical Chemistry and Laboratory Medicine, University Hospital and Faculty of Medicine Carl Gustav Carus, Technische Universität Dresden, Fetscherstrasse 74, 01307 Dresden, Germany; 2 Department of Physiology, Medical School, National and Kapodistrian University of Athens, 75 Mikras Asias Str., 11527 Athens, Greece; 3 Department of Medicine, Columbia University, New York, NY 10032, USA

**Keywords:** non-alcoholic fatty liver disease (NAFLD), non-alcoholic steatohepatitis (NASH), hepatic stellate cells (HSCs), vascular cell adhesion molecule 1 (VCAM-1)

## Abstract

Non-alcoholic fatty liver disease (NAFLD) can progress to non-alcoholic steatohepatitis (NASH), characterized by inflammation and fibrosis. Fibrosis is mediated by hepatic stellate cells (HSC) and their differentiation into activated myofibroblasts; the latter process is also promoted by inflammation. Here we studied the role of the pro-inflammatory adhesion molecule vascular cell adhesion molecule-1 (VCAM-1) in HSCs in NASH. VCAM-1 expression was upregulated in the liver upon NASH induction, and VCAM-1 was found to be present on activated HSCs. We therefore utilized HSC-specific VCAM-1-deficient and appropriate control mice to explore the role of VCAM-1 on HSCs in NASH. However, HSC-specific VCAM-1-deficient mice, as compared to control mice, did not show a difference with regards to steatosis, inflammation and fibrosis in two different models of NASH. Hence, VCAM-1 on HSCs is dispensable for NASH development and progression in mice.

## 1. Introduction

With the continuously expanding obesity pandemic, the prevalence of nonalcoholic fatty liver disease (NAFLD) is constantly increasing [1]. NAFLD is highly associated with insulin resistance, metabolic syndrome and type 2 diabetes and comprises a spectrum of liver pathologies [2,3,4]. Specifically, apart from benign hepatic steatosis, which is characterized by elevated lipid accumulation in hepatocytes, the disease can progress to non-alcoholic steatohepatitis (NASH), characterized by hepatocyte damage, inflammation and fibrosis. NASH affects approximately 1 in 5 NAFLD patients and poses a significantly higher risk for development of cirrhosis and hepatocellular carcinoma (HCC) [5,6]. Since FDA approved treatments for NASH are missing, novel therapeutic strategies are of urgent need [7].

Inflammation is considered a major instigator for the progression of simple steatosis to NASH, with infiltrating monocyte-derived macrophages and activated Kupffer cells playing a cardinal role in this process, via the secretion of inflammatory cytokines and chemokines, such as IL-1b, TNF and CCL-2, as well as major pro-fibrotic mediators, such as TGF-β [8,9,10]. Importantly, these mediators lead to activation of hepatic stellate cells (HSCs), which constitute the principal fibrogenic cell type of the liver. Indeed, upon hepatic damage, HSCs become activated and transdifferentiate into an elongated population of myofibroblasts that produce large amounts of extracellular matrix (ECM) [11]. Continuous HSC activation in response to sustained hepatic damage results to excessive ECM accumulation causing liver fibrosis and scarring, a feature of chronic hepatic disorders including NASH [11].

Despite the multiple soluble mediators, which have been described to activate HSCs in a paracrine fashion provoking their differentiation into myofibroblasts, previous studies have shown that HSCs may also interact with other cells in a direct manner. For instance, HSCs express major histocompatibility molecules (MHC) of both class I and class II, as well as costimulatory molecules, such as CD86 [12,13]. Moreover, pro-inflammatory adhesion molecules, such as VCAM-1, are upregulated in HSCs under inflammatory conditions [14,15,16]. VCAM-1 represents a major counter-receptor for α4β1 integrin in different leukocytes [17,18,19]. In the liver, VCAM-1 in sinusoidal endothelial cells plays a role for leukocyte adhesion during NASH and contributes to fibrosis [20,21].

Interestingly, Lefere et al. reported that serum VCAM-1 levels predicted hepatic fibrosis in patients with NAFLD, indicating a potential role of VCAM-1 in the fibrotic pathogenesis of NASH [22]. Considering the special position of HSCs, which line the space of Disse, and previous findings that VCAM-1 is upregulated in these cells by different inflammatory triggers [14,15,16], we aimed here to investigate the role of VCAM-1 in HSCs for NASH development and progression. To this end, we utilized mice deficient for VCAM-1 in HSCs and appropriate control mice that were subjected to two different established models of diet-induced NASH. Our findings demonstrate that VCAM-1 in HSCs is dispensable for inflammation and fibrosis during NASH.

## 2. Results

### 2.1. VCAM-1 Is Upregulated in the Liver during NASH and Expressed by Activated HSC

**Figure 1:**
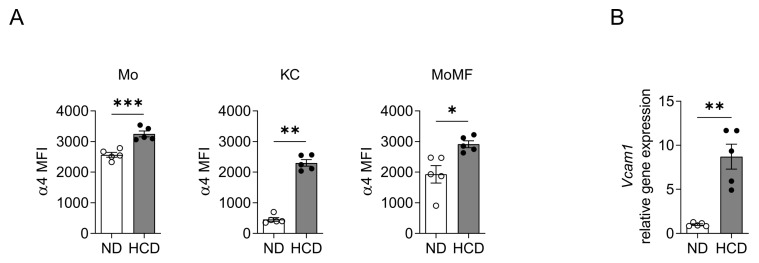
Expression of hepatic VCAM-1 and α4 integrin on monocytes, Kupffer cells and monocyte-derived macrophages during NASH. Wild-type mice were fed a HCD or ND for 6 weeks. (**A**) The expression of α4 integrin (CD49d) was analyzed by flow cytometry. Data are expressed as median fluorescence intensity (MFI) units of α4 integrin on monocytes (Mo, defined as CD11b^+^Ly6G^−^SigF^−^F4/80^−^Ly6C^+^), Kupffer cells (KC, defined as CD11b^+^Ly6G^−^SigF^−^F4/80^+^Ly6C^−^) and monocyte-derived macrophages (MoMF, defined as CD11b^+^Ly6G^−^SigF^−^F4/80^+^Ly6C^+^). (**B**) *Vcam1* mRNA expression in the liver from mice fed a ND or HCD. The expression of *18S* was used for normalization and *Vcam1* expression of ND-fed mice was set as 1. Data are presented as mean ± SEM, *n* = 5 mice/group. * *p* < 0.05, ** *p* < 0.01, *** *p* < 0.001.

Leukocyte integrins have been implicated in fibrotic liver diseases [23]. Previous studies investigating the integrin ligand VCAM-1 focused on the hepatic endothelium [20,21], while only a few studies have mentioned the expression of VCAM-1 in HSCs, without providing any mechanistic evidence on its possible role in HSC function and HSC-related pathophysiology during NAFLD and NASH [14,15]. Therefore, we first fed wild-type mice with a control diet (ND) or a methionine-low, choline-deficient high-fat diet (HCD) for 6 weeks, as described in the Materials and Methods, to induce NASH, and we assessed the expression of α4 integrin, the receptor of VCAM-1, on different leukocyte subpopulations, utilizing flow cytometry. Expression of α4-integrin was upregulated upon NASH induction on monocytes, Kupffer cells and monocyte-derived macrophages (Figure 1A). Moreover, the mRNA expression of VCAM-1 was upregulated in the livers of NASH mice (Figure 1B).

**Figure 2:**
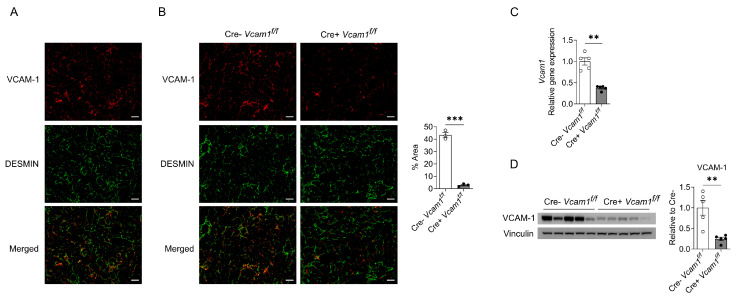
VCAM-1 is expressed in activated HSCs during NASH. (**A**) Wild-type mice were fed a HCD for 6 weeks and immunofluorescence images of liver sections stained for VCAM-1 (red) as well as for the HSC marker desmin (green) are shown. Pseudocolored images are shown; scale bar is 50 µm. (**B**) HSC-specific VCAM-1-deficient mice and control mice (Cre+*Vcam1*^f/f^ and Cre-*Vcam1*^f/f^, respectively) were fed a HCD for 6 weeks. Immunofluorescence staining for VCAM-1 (red) and desmin (green) was performed in liver sections and representative images are shown (left). Pseudocolored images are shown; scale bar is 50 µm. Moreover, quantification is presented (right); data are expressed as the % of VCAM-1 staining-positive area overlapping with desmin staining-positive area per field of vision (*n* = 3 per group). (**C**) *Vcam1* mRNA expression in livers from Cre-*Vcam1*^f/f^ and Cre+*Vcam1*^f/f^ mice fed a HCD for 4 weeks was analyzed by qPCR. *18S* was used for normalization and *Vcam1* expression of Cre-*Vcam1*^f/f^ mice was set as 1 (*n* = 5 per group). (**D**) Immunoblot analysis for VCAM-1 and Vinculin in protein homogenates from livers of Cre-*Vcam1*^f/f^ and Cre+*Vcam1*^f/f^ mice fed a HCD for 4 weeks (left) and quantification of VCAM-1 expression (right) are shown. The results regarding VCAM-1 protein expression are normalized to those of Vinculin and presented as relative to Cre-*Vcam1*^f/f^, set as 1 (*n* = 5 per group). Data are presented as mean ± SEM. ** *p* < 0.01, *** *p* < 0.001.

As HSCs have been previously reported to express VCAM-1, and given its upregulation in the NASH liver, we next sought to investigate the expression and function of VCAM-1 in HSCs during NASH. To study VCAM-1 expression in activated HSCs during NASH, we first performed immunofluorescence stainings for VCAM-1 together with desmin, a marker of activated HSC, in liver sections from wild-type mice subjected to HCD-induced NASH (Figure 2A). Indeed, VCAM-1 showed substantial co-localization with desmin, thus confirming the presence of VCAM-1 in activated HSCs (Figure 2A). In order to further strengthen this finding, we applied the aforementioned staining strategy in liver sections from HSC-specific VCAM-1-deficient mice and control mice with floxed *Vcam-1* (Cre+*Vcam1*^f/f^ and Cre-*Vcam1*^f/f^, respectively) that received the HCD (Figure 2B). Quantification of the immunofluorescence analysis in the stained liver sections revealed that VCAM-1 expression in HSCs was significantly reduced in Cre+*Vcam1*^f/f^ mice as compared to the Cre-*Vcam1*^f/f^ mice (Figure 2B). Thus, this staining corroborated that VCAM-1 is expressed by activated HSCs and confirmed the sufficient deletion of VCAM-1 in HSCs in Cre+*Vcam1*^f/f^ mice (Figure 2B). In addition, both mRNA and protein expression of VCAM-1, as assessed by qPCR and Western Blot analysis, respectively, were significantly reduced in livers of HCD-fed Cre+*Vcam1*^f/f^ mice, as compared to Cre-*Vcam1*^f/f^ mice (Figure 2C,D).

### 2.2. VCAM-1 in HSCs Is Dispensable for NASH Development

**Figure 3:**
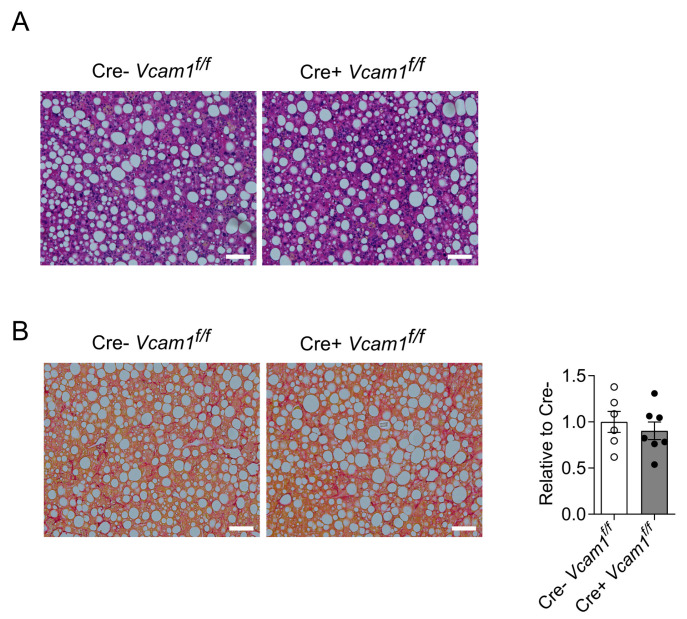
VCAM-1 in HSCs is dispensable for HCD-induced NASH development. HSC-specific VCAM-1-deficient mice and control mice (Cre+*Vcam1*^f/f^ and Cre-*Vcam1*^f/f^, respectively) were fed a HCD for 6 weeks. Representative images of H&E staining (**A**) and Picro Sirius staining (**B**) from liver sections of Cre-*Vcam1*^f/f^ and Cre+*Vcam1*^f/f^ mice are shown. In B (right), quantification of images of Picrosirius staining is shown. The data (percentage of Picrosirius positive area per field of vision) are presented as relative to Cre- mice, set as 1 (*n* = 6–7). Data are presented as mean ± SEM. Scale bars are 100 μm.

Next, in order to study the potential role of VCAM-1 in HSCs for the development and progression of NASH, a comprehensive analysis of the livers of Cre-*Vcam1*^f/f^ and Cre+*Vcam1*^f/f^ mice was performed. Despite the expression of VCAM-1 in activated HSCs in NASH, HSC-specific VCAM-1 deficiency did not affect the grade of steatosis and fibrosis upon HCD feeding (Figure 3A,B).

**Figure 4:**
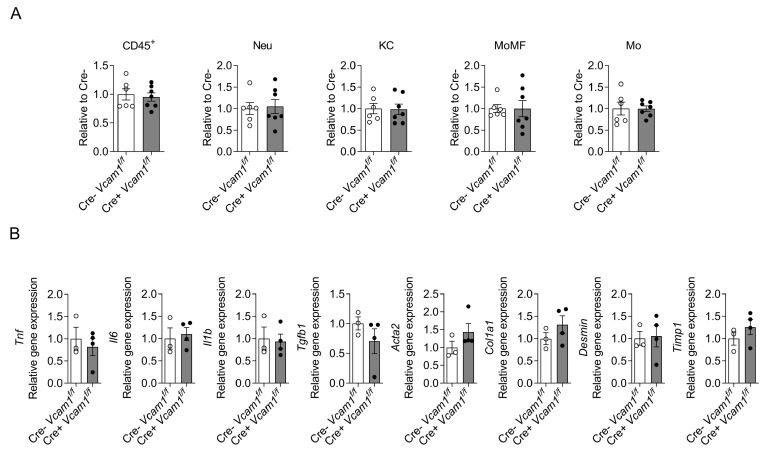
No difference in immune cell populations, inflammation- and fibrosis-related gene expression between HSC-specific VCAM-1-deficient and control mice upon HCD diet. Cre-*Vcam1*^f/f^ and Cre+*Vcam1*^f/f^ mice were fed a HCD for 6 weeks. (**A**) Flow cytometry analysis of cells isolated from the liver of the mice is shown. The number of total leukocytes (defined as CD45^+^ cells), neutrophils (Neu, defined as CD45^+^CD11b^+^Ly6G^+^), Kupffer cells (KC, defined as CD45^+^CD11b^+^Ly6G^−^SigF^−^F4/80^+^Ly6C^−^), monocyte-derived macrophages (MoMF, defined as CD45^+^CD11b^+^Ly6G^−^SigF^−^F4/80^+^Ly6C^+^) and monocytes (Mo, defined as CD45^+^CD11b^+^Ly6G^−^SigF^−^F4/80^−^Ly6C^+^) per gram of liver were analyzed and are presented relative to the numbers from Cre-*Vcam1*^f/f^ mice, set as 1 (*n* = 6–7). (**B**) Inflammation- and fibrosis-related gene expression in the liver of Cre-*Vcam1*^f/f^ and Cre+*Vcam1*^f/f^ mice was analyzed by qPCR. *18S* was used for normalization of mRNA expression and the expression of each gene is presented relative to that of Cre-*Vcam1*^f/f^ mice, set as 1 (*n* = 3–4). Data are presented as mean ± SEM.

To assess NASH-related inflammation, we analyzed leukocyte populations by flow cytometry. No differences between HCD-fed Cre-*Vcam1*^f/f^ and Cre+*Vcam1*^f/f^ mice were observed with regards to the numbers of hepatic total leukocytes, neutrophils, Kupffer cells, monocyte-derived macrophages and infiltrating monocytes, as evaluated by flow cytometry analysis (Figure 4A). Moreover, quantitative PCR analysis of the expression of genes related to inflammation (*Tnf*, *Il6*, *Il1b*) and fibrosis (*Tgfb1*, *Acta2*, *Col1a1*, *Desmin*, *Timp1*) did not reveal any differences due to HSC-specific VCAM-1 deficiency (Figure 4B).

**Figure 5:**
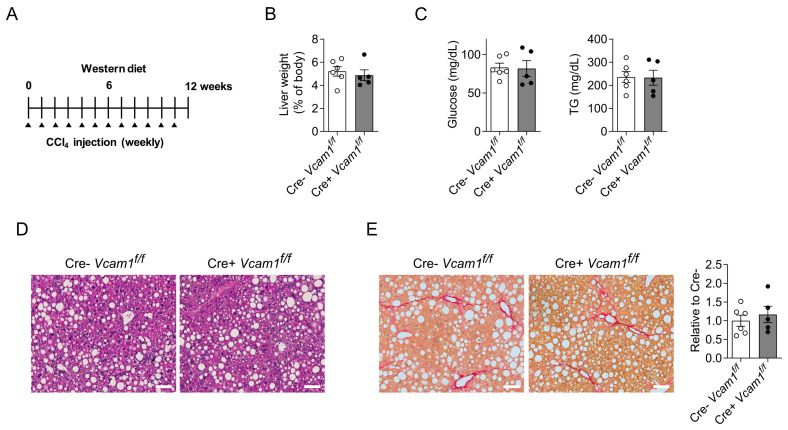
No difference in severity of NASH, induced in a western diet/CCl4 model, between Cre-*Vcam1*^f/f^ and Cre+*Vcam1*^f/f^ mice. (**A**) Experimental scheme for the NASH model used. Cre-*Vcam1*^f/f^ and Cre+*Vcam1*^f/f^ were fed a western diet, accompanied by high fructose- and glucose-containing drinking water and combined with a weekly low dose of carbon tetrachloride (CCl4) administered intraperitoneally for 12 weeks, as described in Materials and Methods. (**B**) Liver weight at the end of the feeding period is shown and expressed as percentage of body weight. (**C**) Levels of fasting blood glucose and triglycerides (TG) after 11 weeks of feeding. (**D**,**E**) Representative images of H&E staining (**D**) and Picrosirius staining (**E**) from liver sections of Cre-*Vcam1*^f/f^ and Cre+*Vcam1*^f/f^ mice are shown. Scale bars are 100 µm. In (**E**) (**right**), quantification of Picrosirius staining is also shown. The data (percentage of Picrosirius positive area per field of vision) are presented as relative to Cre- mice, set as 1. Data are presented as mean ± SEM (*n* = 6 for Cre-*Vcam1*^f/f^ and *n* = 5 for Cre+*Vcam1*^f/f^).

As, in the HCD-NASH model, liver pathology develops owing to choline deficiency in the diet, we next engaged a second model of NASH, in which pathology develops in a different fashion. In particular, we used a 12-week western diet with high sugar supplementation in the water in conjunction with CCl4 administration to accelerate fibrosis development (Figure 5A); this model was recently shown to mimic histological and transcriptomic characteristics of human NASH [24]. There was no difference in liver weight between Cre-*Vcam1*^f/f^ and Cre+*Vcam1*^f/f^ at the end of the feeding period. Systemic metabolism, as assessed by the levels of fasting glucose and triglycerides, was also not different between the two strains (Figure 5B,C). Consistent with the findings from the HCD-model, neither steatosis nor fibrosis was altered in HSC-specific VCAM-1 deficient mice, as compared to the control mice (Figure 5D,E).

**Figure 6:**
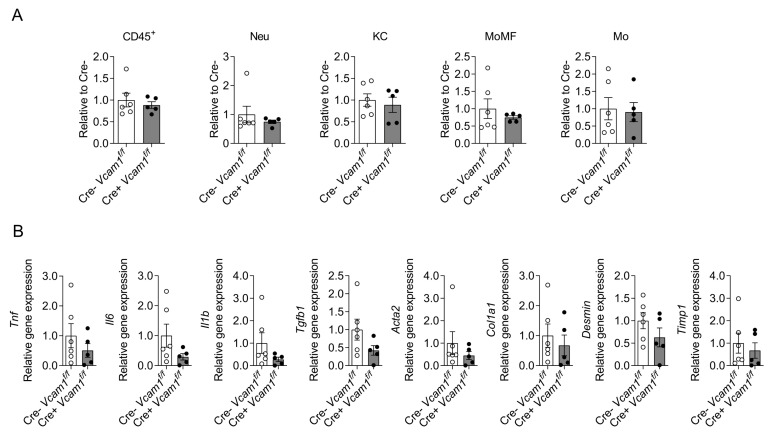
HSC-specific VCAM-1-deficiency is not linked to altered hepatic inflammation during western diet- and CCl4 –induced NASH. Cre-*Vcam1*^f/f^ and Cre+*Vcam1*^f/f^ were fed a western diet, accompanied by high fructose- and glucose-containing drinking water and combined with a weekly low dose of carbon tetrachloride (CCl4) administered intraperitoneally for 12 weeks, as described in Materials and Methods and shown in Figure 5A. (**A**) Flow cytometry analysis of cells isolated from the liver of the mice is shown. The number of total leukocytes (defined as CD45^+^ cells), neutrophils (Neu, defined as CD45^+^CD11b^+^Ly6G^+^), Kupffer cells (KC, defined as CD45^+^CD11b^+^Ly6G^−^F4/80^+^Ly6C^−^), monocyte-derived macrophages (MoMF, defined as CD45^+^CD11b^+^Ly6G^−^F4/80^+^Ly6C^+^) and monocytes (Mo, defined as CD45^+^CD11b^+^Ly6G^−^F4/80^−^Ly6C^+^) per gram of liver were analyzed and are presented relative to the numbers from Cre-*Vcam1*^f/f^ mice, set as 1. (**B**) Inflammation- and fibrosis-related gene expression in the liver of Cre-*Vcam1*^f/f^ and Cre+*Vcam1*^f/f^ mice was analyzed by qPCR. *18S* was used for normalization of mRNA expression and the expression of each gene is presented as relative to that of Cre-*Vcam1*^f/f^, set as 1. *n* = 6 for Cre-*Vcam1*^f/f^ and *n* = 5 for Cre+*Vcam1*^f/f^. Data are presented as mean ± SEM.

Moreover, analysis of the inflammatory milieu of the liver of Cre-*Vcam1*^f/f^ and Cre+*Vcam1*^f/f^ mice by flow cytometry displayed no differences in the numbers of hepatic total leukocytes, neutrophils, Kupffer cells, monocyte-derived macrophages and infiltrating monocytes (Figure 6A). Furthermore, expression of genes related to inflammation (*Tnf*, *Il6*, *Il1b*) and fibrosis (*Tgfb1*, *Acta2*, *Col1a1*, *Desmin*, *Timp1*) was also not affected by HSC-specific VCAM-1 deficiency (Figure 6B). Together, VCAM-1 in HSCs does not contribute to liver steatosis, inflammation or fibrosis development in the course of NAFLD/NASH, as assessed in two different experimental models.

## 3. Discussion

HSCs are the cellular mediators of fibrosis during NASH via their differentiation from their quiescent state to activated HSCs and myofibroblasts [9,25]. Their activation is mediated by soluble mediators such as IL-1 and TNF, secreted by hepatocytes and several populations of immune cells, as well as major fibrosis-promoting factors such as TGF-β, expressed mainly by activated macrophages, both Kupffer cells and infiltrating monocyte-derived macrophages [9,26,27]. In contrast, less information exists about the role adhesion receptors, such as VCAM-1, may play in the context of HSC activation and transdifferentiation into myofibroblasts, although previous studies have reported upregulation of VCAM-1 in activated HSCs [14,15,16].

This prompted us to study the role of VCAM-1 in HSCs during NASH. We hypothesized that VCAM-1 in HSCs could regulate the accumulation of leukocyte populations in the liver microenvironment during NASH, or regulate intracellular signaling processes involved in HSC transdifferentiation into myofibroblasts. In line with this hypothesis, we have previously shown that adhesion of macrophages onto adipocytes, which are also cells of mesenchymal origin, in a manner that involved adipocyte VCAM-1 expression, can modulate their functional properties [19]. Herein, we first observed an upregulation of VCAM-1 expression in livers from NASH mice as compared to control mice, accompanied by upregulation of α4 integrin, the receptor of VCAM-1, on monocytes, Kupffer cells and monocyte-derived macrophages. By immunofluorescence analysis of liver sections we confirmed the presence of VCAM-1 in HSCs, utilizing desmin as a pan-HSC marker. It should be noted that other markers, such as a-SMA, which is specific for activated-HSCs, were not used in the present study. However, as the model of HCD-induced NASH in mice displays extensive liver fibrosis [21,28,29,30], the majority of HSCs have likely acquired an activated state; hence, our co-staining of liver sections for VCAM-1 and the pan-HSC marker desmin suggests the presence of VCAM-1 on activated HSCs. Previous studies have reported an upregulation of VCAM-1 in the liver and specifically in HSCs under inflammatory conditions, e.g., upon LPS administration or CCl4-induced fibrosis [14,15,16]. Importantly, TLR-4 activation of HSCs led to VCAM-1 upregulation [16]. However, the function of VCAM-1 in HSCs was not studied under NASH conditions so far.

Despite the interesting finding that VCAM-1 expression in HSCs was enhanced during NASH, HSC-specific VCAM-1 deficient mice did not display any differences in steatosis, inflammation and fibrosis, compared to the control mice, as assessed by histology, flow cytometry and gene expression studies in the HCD-induced model. The HCD model is nowadays widely utilized for NASH studies [21,28,29,30]. We further confirmed our findings by subjecting HSC-specific VCAM-1 deficient and control mice to a second model of NASH induction, which is of longer duration as compared to the HCD, while mimicking several aspects of human NASH [24]. The absence of difference in the phenotype of Cre+*Vcam1*^f/f^ mice as compared to the Cre-*Vcam1*^f/f^ ones upon NASH induction in the latter model confirmed that VCAM-1 in HSC is dispensable for the progression of the disease. It is possible that other adhesion molecules expressed in HSCs may have compensated for the absence of HSC VCAM-1 in the Cre+*Vcam1*^f/f^ mice; thus a potential function of HSC VCAM-1 in NASH cannot be entirely excluded. Additionally, we can conclude that HSC VCAM-1 is dispensable for disease development and progression only in the two NASH models used. We cannot exclude that HSC VCAM-1 could play a role in a NASH or liver fibrosis model different from the two models used here. On the contrary, VCAM-1 on LSEC has a role for the accumulation of leukocytes during NASH, thereby accelerating hepatic inflammation and the progression of the disease [20,21].

Although serum VCAM-1 levels correlate with hepatic fibrosis in patients with NAFLD [22], a finding that may be linked with the upregulation of VCAM-1 in activated HSCs, as identified here, our functional results suggest that VCAM-1 in HSCs does not play a pathophysiological role in fibrosis progression. Hence, future studies should interrogate the utilization of VCAM-1 as a biomarker for NASH progression. Moreover, while no function of HSC VCAM-1 in liver fibrosis was found here, we cannot exclude that VCAM-1 in other cells could be a therapeutic target in NASH. These aspects should be addressed in future studies.

## 4. Materials and Methods

### 4.1. Animal Studies

Wild-type mice (C57BL/6) were from Charles River (Sulzfeld, Germany). Hepatic stellate cell-specific deletion of *Vcam1* was achieved by crossing mice carrying a floxed *Vcam1* allele (Jackson Laboratories, Bar Harbor, ME, USA) with mice in which Cre recombinase expression is driven by the mouse Lecithin:retinol acyltransferase (LRAT) promoter [31]. Wild-type mice were fed a normal chow diet (ND) as control or fed a methionine-low, choline-deficient high-fat diet (HCD, 60% kcal from fat, 0.1% methionine, A06071302, Research Diets) [28,29,32]. Lrat-Cre negative *Vcam1* floxed/floxed and Lrat-Cre positive *Vcam1* floxed/floxed mice (designated Cre-*Vcam1*^f/f^ and Cre+*Vcam1*^f/f^, respectively) were fed the HCD.

In other experiments, Cre-*Vcam1*^f/f^ and Cre+*Vcam1*^f/f^ mice were fed a western diet, specifically a high fat, high fructose, and high cholesterol diet (21.1% fat, 41% sucrose, and 1.25% cholesterol, Teklad diets, TD. 120528) together with water including high sugar concentrations [23.1 g/L D-Fructose (SERVA, Heidelberg, Germany, 21830) and 18.9 g/L D-Glucose (Sigma-Aldrich, Taufkirchen, Germany, G8270)] for 12 weeks. In addition, the mice received weekly an intraperitoneal low dose of carbon tetrachloride (CCl4, Sigma-Aldrich, 289116, 0.32 µg/g of body weight) as an accelerator of liver fibrosis [24]. After 11 weeks of feeding and upon overnight fasting, blood glucose levels were measured in tail vein blood samples with a glucose meter device (Accu-Chek, Roche, Vienna, Austria), while the levels of triglycerides were measured with an Accutrend Plus system (Roche).

Mice were housed on a standard 12 h light/12 h dark cycle under specific pathogen-free conditions. Eight to ten week old male mice were used in experiments. At the end of the feeding period, mice were euthanized, undergoing also systemic perfusion with phosphate-buffered saline (PBS), and tissues were collected for further analysis. Animal experiments were approved by the Landesdirektion Sachsen, Germany and by the Region of Attica, Greece.

### 4.2. Histological Analysis

Mouse livers were isolated from euthanized mice and fixed with 4% PFA for 24 h. For Hematoxylin and Eosin (H&E) staining, liver samples were embedded in paraffin, and cut liver sections were deparaffinized and rehydrated. The sections were stained with Mayers Haematoxylin (SAV, Liquid Production GmbH, Flintsbach am Inn, Germany, 10231.02500) and Eosin (Klinikapotheke Universitätsklinikum, Dresden, Germany) and mounted with VectaMount (Vector Laboratories, Newark, CA, USA, H-5000-60) after a series of ethanol washings (80%, 95%, 100%). For Picrosirius red staining, deparaffinized and rehydrated liver sections were stained with Picrosirius red solution (Sigma-Aldrich, 365548) for 1 h and then washed with 1% acetic acid. The liver sections were mounted with VectaMount after a series of ethanol washing as before. Images were acquired utilizing a ZEISS Axio Observer Z1-computerized microscope and Picrosirius red positive areas per field of vision were quantified from at least 12 images per mouse using the Fiji software (ImageJ 2.1.0/1.53c).

For immunofluorescence staining, fixed liver samples were embedded in OCT upon incubation with a series of sucrose solutions (10%, 20%, 30%) to achieve tissue cryoprotection. Liver sections were dried and permeabilized with 0.1% Triton X-100 and then blocked using a serum-free protein block solution (Dako, Waldbronn, Germany, X090930-2). Liver sections were then incubated with primary antibodies against VCAM-1 (1:10, eBioscience, Darmstadt, Germany, # 14-1061-85) and desmin (1:100, Abcam, Berlin, Germany, ab32362) overnight at 4 °C. After washing with PBS, sections were incubated with secondary antibodies, namely Donkey anti-Rat (H + L) Alexa Fluor 555 (Abcam, ab150150) and Donkey anti-rabbit (H + L) Alexa Fluor 647 (Invitrogen, Darmstadt, Germany, A-31573) for 90 min at RT and counterstained with DAPI (Sigma-Aldrich, D9542). To reduce tissue autofluorescence, sections were incubated with TrueBlack^®^ Lipofuscin Autofluorescence Quencher (Biotium, Fremont, CA, USA, #23007) for 30 s and mounted. Images were acquired with a ZEISS Axio Observer Z1 computerized microscope equipped with the Zen 3.2 (Blue edition) software. Images are shown in pseudocolor; the display color of the channels was set as to optimize clarity of merged images.

### 4.3. Flow Cytometry Analysis

The left lobe of the liver was isolated, minced and digested in high glucose DMEM containing 0.5% BSA, collagenase D (1.5 mg/mL, COLLD-RO, Roche), and DNaseI (5U/mL, 04716728001, Roche) for 1 h at 37 °C with shaking. The cell suspension was filtered through a 100 µm cell strainer and centrifuged at 600× *g* for 7 min at 4 °C. Afterwards the red blood cells were lysed using RBC Lysis Buffer (eBioscience, 00-4300-54) for 5 min at room temperature. Additionally, cell debris were removed by utilizing a debris removal solution (Miltenyi Biotec, Bergisch Gladbach, Germany, 130-109-398).

For analysis of α4 integrin expression in hepatic immune cells, upon debris removal the cells were incubated with mouse CD45 Microbeads (Miltenyi Biotec, 130-052-301) for 15 min at 4 °C and CD45^+^ cells were collected by LS column (Miltenyi Biotec, 130-042-401). Then, they were stained with antibodies against CD11b (M1/70, Biolegend, San Diego, CA, USA, 101230), SiglecF (E50-2440, BD Biosciences, Heidelberg, Germany, 562680), Ly6G (1A8, Biolegend, 127624), F4/80 (BM8, eBioscience, 25-4801-82), Ly6C (AL-21, BD Biosciences, 553104), α4 integrin/CD49d (9C10, Biolegend, 103706), and Hoechst 33258 (Invitrogen, H1398). For the analysis of hepatic innate immune cells derived from livers of HCD-fed Cre-*Vcam1*^f/f^ and Cre+*Vcam1*^f/f^ mice, CD45^+^ cells, isolated as described above, were stained with antibodies against CD11b (M1/70, Invitrogen, 12-0112-82), SiglecF (E50-2440, BD Biosciences, 562680), Ly6G (1A8, Biolegend, 127624), F4/80 (BM8, eBioscience, 25-4801-82), Ly6C (AL-21, BD Biosciences, 553104), CD45 (30-F11, Biolegend, 103130), and Hoechst 33258 (Invitrogen, H1398). Stained cells were analyzed on a BD FACSCanto™ II cytometer (BD Biosciences) and analyzed by FlowJo software (v10.1r7).

For analyzing the hepatic innate immune cells acquired from Cre-*Vcam1*^f/f^ and Cre+*Vcam1*^f/f^ mice, which were fed a western diet combined with CCl4 treatment, upon debris removal, the cells were stained with antibodies against CD45 (30-F11, Biolegend, 103133), CD11b (M1/70.15, Invitrogen, RM2804), Ly6G (1A8, BD Biosciences, 560599), F4/80 (BM8, eBioscience, 25-4801-82), Ly6C (AL-21, BD Biosciences, 553104). Stained cells were run on an ARIA III cytometer (BD Biosciences) and analyzed by FlowJo software.

### 4.4. Gene Expression Analysis

Liver tissues were snap frozen in liquid nitrogen or kept in RNAlater (Invitrogen, AM7020). The liver samples were homogenized in TriReagent (MRC, Cincinnati, OH, USA, TR 118) by using the Precellys 24 tissue homogenizer and after phase separation, the RNA was precipitated using 75% ethanol. Finally, RNA was isolated by NucleoSpin^®^ RNA kit (Macherey-Nagel, Dueren, Germany, 740955.250) and reverse-transcribed with the iScript cDNA Synthesis Kit (Bio-Rad, Feldkirchen, Germany, 1708890). The qPCR was performed utilizing the SsoFast^™^ EvaGreen^®^ Supermix (Bio-Rad, 1725204) and gene-specific primers on a CFX384 Real-time PCR detection system (Bio-Rad). Relative mRNA expression levels were calculated according to the ΔΔCt method upon normalization to *18S* [33]. The primers used in this study are:

*Vcam1* (F:CTTCCCAGAACCCTTCTCAG, R:GGGACCATTCCAGTCACTTC)
*Tnf* (F:AGCCCCCAGTCTGTATCCTTCT, R:AAGCCCATTTGAGTCCTTGATG),
*Il1b* (F:ATCCCAAGCAATACCCAAAG, R:GTGCTGATGTACCAGTTGGG),
*Il6* (F:CCTTCCTACCCCAATTTCCAAT, R:AACGCACTAGGTTTGCCGAGTA),
*Tgfb1* (F:CACAATCATGTTGGACAACTGCTCC, R:CTTCAGCTCCACAGAGAAGAACTGC),
*Col1a1* (F:GAGCGGAGAGTACTGGATCG, R:GCTTCTTTTCCTTGGGGTTC),
*Desmin* (F:GTGGATGCAGCCACTCTAGC, R:TTAGCCGCGATGGTCTCATAC),
*Acta2* (F:ACTGGGACGACATGGAAAAG, R:GTTCAGTGGTGCCTCTGTCA)
*Timp1* (F:TACACCCCAGTCATGGAAAGC, R:CGGCCCGTGATGAGAAACT)
*18S* (F:GTTCCGACCATAAACGATGCC, R:TGGTGGTGCCCTTCCGTCAAT)

### 4.5. Immunoblot Analysis

Liver tissues were snap frozen in liquid nitrogen and homogenized in RIPA lysis buffer (Santa Cruz, Heidelberg, Germany, sc-24948A) containing a protease and phosphatase inhibitor cocktail (Roche, 04693159001, CO-RO) by using the Precellys evolution homogenizer (Bertin Technologies, Frankfurt am Main, Germany) and then centrifuged at 14,000× *g* for 20 min at 4 °C. The supernatant was collected and protein concentrations were determined by using a BCA protein assay kit (Thermo Scientific, Darmstadt, Germany, 23227). The protein samples (30 µg) were separated on a NuPAGE™ 4–12% gel (Thermo Scientific, NP0323BOX) and transferred to a PVDF membrane (Bio-Rad, 1620177). The membrane was blocked with 5% skim milk for 1 h at RT and then incubated with primary antibody against VCAM-1 (Abcam, ab134047) overnight at 4 °C, followed by incubation with appropriate secondary antibody. After membrane stripping using a Restore Western Blot Stripping-Buffer (Thermo Scientific, 21059), the membrane was blocked again with 5% skim milk for 1 h at RT and then incubated with antibody against Vinculin (Cell signalling, Leiden, The Netherlands, 4650) overnight at 4 °C, followed by incubation with appropriate secondary antibody. Detection in each case was performed with The Super Signal West Pico PLUS Chemiluminescent substrate (Thermo Scientific, 34579) in a VILBER imaging system (FUSION FX, Eberhardzell, Germany). Densitometry was performed by using the Fiji software.

### 4.6. Statistical Analysis

A two-tailed Student’s *t*-test or a Mann–Whitney U test was used. The Graph Pad Prism 8 software was used and significance was set at *p* < 0.05.

## Data Availability

The data are available upon reasonable request from the corresponding authors.
